# CORNAS: coverage-dependent RNA-Seq analysis of gene expression data without biological replicates

**DOI:** 10.1186/s12859-017-1974-4

**Published:** 2017-12-28

**Authors:** Joel Z. B. Low, Tsung Fei Khang, Martti T. Tammi

**Affiliations:** 1 Institute of Biological Sciences, Faculty of Science, University of Malaya, Kuala Lumpur, 50603 Malaysia; 2 Institute of Mathematical Sciences, Faculty of Science, University of Malaya, Kuala Lumpur, 50603 Malaysia; 3 University of Malaya Centre for Data Analytics, University of Malaya, Kuala Lumpur, 50603 Malaysia; 4 Sime Darby Technology Centre Sdn. Bhd., UPM-MTDC Technology Centre III, University Putra Malaysia, Serdang, 43400 Malaysia

**Keywords:** RNA-Seq, Unreplicated experiments, Bayesian statistics, Differential gene expression, Sequencing coverage, Illumina

## Abstract

**Background:**

In current statistical methods for calling differentially expressed genes in RNA-Seq experiments, the assumption is that an adjusted observed gene count represents an unknown true gene count. This adjustment usually consists of a normalization step to account for heterogeneous sample library sizes, and then the resulting normalized gene counts are used as input for parametric or non-parametric differential gene expression tests. A distribution of true gene counts, each with a different probability, can result in the same observed gene count. Importantly, sequencing coverage information is currently not explicitly incorporated into any of the statistical models used for RNA-Seq analysis.

**Results:**

We developed a fast Bayesian method which uses the sequencing coverage information determined from the concentration of an RNA sample to estimate the posterior distribution of a true gene count. Our method has better or comparable performance compared to NOISeq and GFOLD, according to the results from simulations and experiments with real unreplicated data. We incorporated a previously unused sequencing coverage parameter into a procedure for differential gene expression analysis with RNA-Seq data.

**Conclusions:**

Our results suggest that our method can be used to overcome analytical bottlenecks in experiments with limited number of replicates and low sequencing coverage. The method is implemented in CORNAS (Coverage-dependent RNA-Seq), and is available at https://github.com/joel-lzb/CORNAS.

**Electronic supplementary material:**

The online version of this article (doi:10.1186/s12859-017-1974-4) contains supplementary material, which is available to authorized users.

## Background

Large-scale mining of gene signatures that are significantly associated with specific phenotype classes is a commonly desired outcome from transcriptome analyses. RNA-sequencing (RNA-Seq) has become the tool of choice for gene expression profiling, complementing the traditional microarray in several important aspects: it samples the transcriptome more thoroughly, detects isoforms, and works without prior knowledge of the target transcriptome [1, 2]. Since the publication of the first RNA-Seq paper [3], extensive interest in RNA-Seq has resulted in the rapid development and deployment of sequencing platforms such as 454, Illumina and Solexa. These platforms naturally spurred concurrent development of data processing and analysis methods to extract biological meaning from RNA-Seq data.

A typical RNA-Seq data analysis begins with choosing reads that pass quality control criteria, mapping them to a reference genome, and then quantifying the gene counts. After normalization, the resulting data matrix is ready for statistical analysis, for which a bewildering number alternative methods are available [4, 5]. Regardless of whether these are parametric (e.g. DESeq [6], EdgeR [7], DEGSeq [8], BaySeq [9]) or nonparametric (e.g. NOISeq [10], SAMSeq [11]), in all of them the underlying assumption is that the observed gene counts are adequate representations of the actual true gene counts.

In genome sequencing, the ratio of total read length to genome size provides a coverage measure that is important for evaluating the completeness of an assembled genome. Extending the concept of coverage for transcriptome size is, however, not straightforward. Firstly, transcriptome sizes vary between different tissue types in the same organism, and even between cells of the same tissue type [12]. Next, the relative proportions of mRNA species between cells can be highly variable [13]. For example, in genetically identical yeast cells, variation of more than 800 copies of an mRNA species per cell has been observed [14].

For accurate quantification of 95% of transcripts in a human cell line, up to 700 million (M) reads are needed [15]. In contrast, RNA-Seq experiments often produce reads much less than 700M [16], low enough for stochastic effects to have a large impact on an interpretation of statistical analysis results. Without substantial decrease in sequencing cost, researchers are often forced to prioritize the increase in number of replicates over total number of reads, since this is the best strategy to increase statistical power for differential gene expression analysis [17]. Several challenges then arise. Assuming perfect read-mapping and quantification, it is unclear whether observed gene counts are representative of the true gene counts, since a large range of true gene counts could have produced a particular observed gene count due to a strong stochastic effect. Compounding the problem are biases inherent in technical RNA-Seq library preparation and sequencing [18], problems that are only recently receiving serious attention [19].

We have developed CORNAS (COverage-dependent RNA-Seq), a Bayesian method to infer the posterior distribution of a true gene count. The novelty of this method is that it incorporates a coverage parameter determined from RNA sample concentration. Subsequently, the comparison of posterior distributions of true gene counts provides a basis for calling differentially expressed genes (DEG). We report the application of CORNAS in unreplicated RNA-Seq experiments and discuss the prospect of its use in overcoming the analytical limitations of such experiments.

## Results

### Definition of true gene count and sample coverage

We first define the true gene count as the total number of mRNA copies of a gene, in a sample prepared for a sequencing run. This definition holds for a sample containing single or multiple cells. This value cannot be known with certainty solely from the observed gene count, since the latter can, in principle, be derived from multiple different true gene counts. However, information about sample coverage can improve the process of estimating the true gene count.

The coverage of a sample (*b*) is defined as the number of cDNA fragments sequenced (*S*) divided by the total cDNA fragment population size (*N*). Single-end sequencing produces one read to represent one cDNA sequenced, while paired-end sequencing produces two reads to represent one cDNA sequenced.

The calculation of sample coverage in the context of the Illumina sequencing protocol can be based on mRNA sample concentration. We reason that the amount of cDNA produced at the step prior to PCR provides the key to a reasonable estimate of sample coverage because: 1) the fragmentation step during sample library preparation causes homogeneity of the cDNA molecule sizes (500 bp); 2) the volume and concentration after PCR is known (40 *μ*L of 200nM cDNA) and; 3) the number of PCR cycles is known (14 cycles). The cDNA fragments undergo PCR to improve the chance of getting at least a sequencing coverage of one. Assuming perfect amplification efficiency, each cDNA fragment is amplified 2^14^ times during PCR. Thus, the number of cDNA fragments prior to PCR is estimated as 4.818×10^12^/ 2^14^≈300 M (details in Additional file 1). We use this quantity as the estimated total fragment population size to determine coverage, since it most closely resembles the mRNA amount we started off with.

### Chance mechanism generating a Generalized Poisson distribution for observed gene counts

When cDNA fragments are loaded into a sequencing run, short reads are assumed to be generated randomly from the loaded cDNA fragments. Thus, a true gene count induces a probability distribution of observed gene count. To find a probabilistic model that best describes the latter, we made a series of simulations to determine the mean-variance relationship of the observed gene counts under six coverage values: 0.5, 0.4, 0.25, 0.1, 0.01 and 0.001. These coverages were computed assuming that 150M, 120M, 75M, 30M, 3M and 0.3M reads were respectively sequenced from a total fragment population size of 300M. For each coverage, we generated an empirical distribution of the observed counts for true count values ranging from 1 to 100,000 (details in the “Methods” section).

The simulation results provided three important observations: the mean of observed counts is proportional to the coverage, underdispersion occurs (i.e. variance less than mean) with increasing coverage (Additional file 1: Figure S1), and a linear model adequately describes the relationship between the mean-variance ratio and coverage (Eq. 6). These results suggest that the Generalized Poisson (GP) distribution [20] is suitable for modelling the distribution of observed gene counts (*X*) given a true gene count (*T*). The probability mass function of the GP with parameters *λ*_1_ and *λ*_2_ is given by

1 $$ P(X=x|T=k) = \frac{\lambda_{1}(\lambda_{1}+x\lambda_{2})^{x-1} e^{-(\lambda_{1}+x\lambda_{2})}} {x!} \,  $$

where *x*=0,1,2,…, *λ*_1_>0, and |*λ*_2_|<1. Its mean and its variance are given by

$$\begin{array}{@{}rcl@{}} \mathbb{E}(X|T) &=& \lambda_{1} / (1-\lambda_{2}) \,\\ \text{Var}(X|T) &=& \mathbb{E}(X|T) / (1-\lambda_{2})^{2} \, \end{array} $$

implying that $\lambda _{2} = 1- \sqrt {m}$, where *m* is the mean-variance ratio (0<*m*<4). The mean of the observed gene count given the true gene count is proportional to the product of the coverage *b* and the true count *k*, giving $\lambda _{1} = bk\sqrt {m}$. The Poisson distribution with mean *λ*_1_ is a special case of the GP when *m*=1.

### A Bayesian model for estimating true gene counts given observed gene counts and sequencing coverage

The importance of the GP model in Eq. 1 stems from the fact that reverse conditioning enables us to consider the probability distribution of the true gene count (*T*) given an observed gene count and sequencing coverage (i.e. the posterior distribution of the true gene count). Let us assume a uniform prior distribution for *T* over values of 1,2,…. Application of Bayes Theorem yields:

2 $$\begin{array}{*{20}l}{} P(T=k|X=x) &= \frac{P(X=x|T=k)}{\sum\limits_{j=x}^{\infty} P(X=x|T=j)} \\ &= \frac{k(bk\sqrt{m}+x(1-\sqrt{m}))^{x-1}e^{-bk\sqrt{m}}} {\sum\limits_{j=x}^{\infty} j(bj\sqrt{m}+x(1-\sqrt{m}))^{x-1}e^{-bj\sqrt{m}}} \, \end{array} $$

where *k*≥*x*. Note that although we used an improper prior, the resulting posterior distribution is proper. Interestingly, the gamma distribution provides a good approximation to Eq. 2 (see [21] for mathematical proof). Here, we found that the approximation is excellent if the mean *μ* and the variance *σ* ^2^ of the gamma distribution relates to the coverage *b* and the observed gene count *x* as (Additional file 1: Figure S2):

3 $$ \mu \approx \frac{x+1}{b} - \left(1 + \frac{1}{2b}\right)^{-1} ;   $$

4 $$ \sigma^{2} \approx \frac{x+1}{\left[b(b+1)\right]^{2}}.   $$

Thus the probability density of the approximating gamma distribution is given by

5 $$ f(k|x) = \frac{1}{\Gamma(\alpha) \beta^{\alpha}} k^{\alpha - 1} e^{-k/\beta},  $$

where *k*≥0, *α*=*μ* ^2^/*σ* ^2^ and *β*=*σ* ^2^/*μ*. The approximation provides a computationally efficient means to calculate the cumulative distribution function of the posterior distribution of the true gene count.

**Fig. 1:**
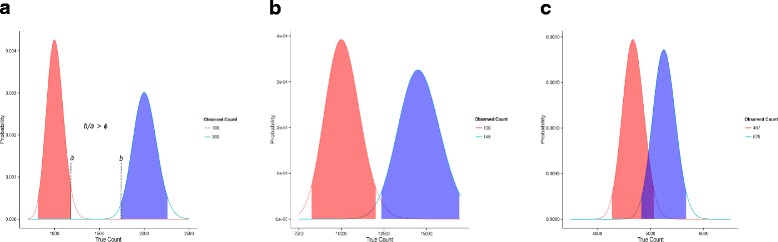
Illustration of how DEG calls are made in CORNAS. By default, the fold-change (*ϕ*) is 1.5. **a** A DEG, **b** Not a DEG, **c** Not a DEG

A statistical test for calling differentially expressed genes in the case of unreplicated RNA-Seq experiments can be based on the posterior distribution of the true gene count (Eq. 2) as follows. For a single control and a single treatment sample, if we have information about sequencing coverage for the control sample (*b*_0_) and the treatment sample (*b*_1_), then, given the observed gene count for the control (*x*_0_) and the treatment (*x*_1_) group, the posterior distribution of their true gene count is approximately gamma (Eq. 5). We declare a gene to be differentially up-regulated in the treatment group if the latter has a larger posterior mean, and its 0.5th percentile is at least 1.5 fold (default) larger than the 99.5th percentile of the control group. Conversely, a gene is differentially down-regulated in the treatment group if the latter has a smaller posterior mean, and its 99.5th percentile is at least 1.5 fold (default) smaller than the 0.5th percentile of the control group (Fig. 1). This procedure is fast because the percentiles of the gamma distribution are easily computed. Furthermore, declaring genes to be differentially expressed using this procedure implies there is a 0.995^2^≈0.99 probability that the true gene count in the two samples differ by at least 1.5 fold.

### Performance evaluation of CORNAS

We conducted a series of tests comparing the performance of CORNAS against NOISeq [10] and GFOLD [22] using both simulated and real data sets. We chose GFOLD and NOISeq, because both have been reported to return relatively small number of false positives among the genes flagged as differentially expressed when applied to unreplicated RNA-Seq data sets compared to other popular methods such as DESeq2 and edgeR [5].

#### Test 1: detection of differentially expressed genes in simulated true gene count data

We tested CORNAS using four coverages: 0.5, 0.25, 0.1 and 0.01, on simulated true gene counts ranging from 1 to 10,000. The relative frequency of calling differentially expressed genes (DEG) was recorded in 100 independent trials for the scenario of no-fold change (no effect), 1.5-fold change (weak effect) and 2-fold change (strong effect) between control and treatment. The false positive rate (FPR) was estimated as the DEG call rate in the scenario of no-fold change. The true positive rate (TPR), or sensitivity, is the DEG call rate in the weak and strong effect scenarios.

**Fig. 2:**
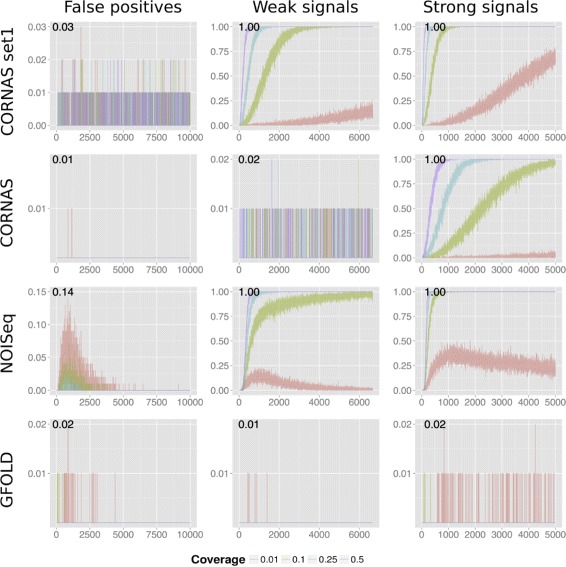
DEG detection using simulated true count data. The Y-axis is the proportion of DEG called in 100 replicates. The X-axis is the true count of Sample 1. Comparison is made against Sample 2, which either has the same (False positives), 1.5 times more (Weak signals), or 2 times more (Strong signals) true counts. The numbers at the top left of each plot denotes the Y-axis maximum. The maximum true counts for false positive, weak signal and strong signal conditions are 10,000, 6,666 and 5,000 respectively. CORNAS set1 refers to CORNAS with *ϕ*=1, while CORNAS refers to the default *ϕ*=1.5

In general, we observed decreased false positives and increased DEG call rates with increasing coverage and increasing number of true gene counts (Fig. 2). Compared to GFOLD and CORNAS default, NOISeq produced the largest FPR when true gene counts are low. NOISeq’s sensitivity is generally good except at low coverage of 0.01; its DEG call rate begins to fall when true counts are over 1,000. GFOLD showed very low sensitivity, which is consistent with its conservative behaviour reported in [5]. CORNAS showed excellent control of FPR and a dependence on the fold change threshold for detecting DEG under weak and strong signal scenarios. For example, CORNAS default (*ϕ*=1.5) performed very poorly under the weak signal scenario, so that if the detection of such genes is of interest then *ϕ* should be adjusted to a lower value such as 1 (CORNAS set1). In general, the sensitivity of CORNAS increases with larger true count, and converges to 1 quickly for coverage values of 0.1 or more.

#### Test 2: compcodeR simulation

The distribution of observed gene counts is popularly modelled using the negative binomial distribution, and the compcodeR R package [23] provides a simulator for simulating RNA-Seq count data based on this distribution. Gene lengths were assumed to be equal and set at 1000 bases. We used the example provided in [23] to create a control-treatment comparison (five replicates in each group) with 624 up-regulated genes and 625 down-regulated genes in the control group for a simulated transcriptome of 12,498 genes. From this data matrix, a total of 25 unreplicated data sets were constructed. For CORNAS, we evaluated the outcome of two different coverages on the sample comparisons; one estimated at 10 times less than compcodeR coverage (CORNAS_10xless), and another at 100 times less (CORNAS_100xless) (Supporting Data). We made two separate NOISeq runs, one without length normalization (NOISeq_nln), and another using the trimmed mean of M-values normalization (NOISeq_tmmnl).

**Fig. 3:**
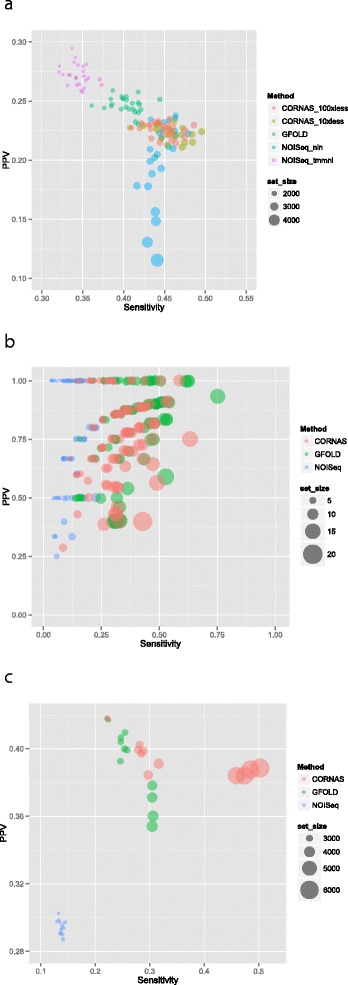
Scatterplots of PPV against sensitivity. The size of each dot is proportional to the DEG set size. **a** compcodeR simulation, **b** Human sex-specific gene expression, **c** Human tissue-specific gene expression

Positive predictive value (PPV) and sensitivity were low for all methods; nonetheless, CORNAS showed relatively greater sensitivity than the other methods, whereas GFOLD had relatively better PPV (Fig. 3a). The F-scores for all methods were very similar (Table 1). CORNAS called a larger DEG set size compared to other methods. Unlike NOISeq_nln, the larger DEG set size called by CORNAS did not substantially reduce its PPV. Both CORNAS_100xless and CORNAS_10xless showed similar performance.

**Table 1:** The mean F-score calculated for each method for Test 2, Test 3 and Test 4 cases

Method	F-score
**Test 2**	
GFOLD	0.31
NOISeq_tmmnl	0.30
CORNAS_100xless	0.30
CORNAS_10xless	0.30
NOISeq_nln	0.28
**Test 3**	
GFOLD	0.51
CORNAS	0.45
NOISeq	0.22
**Test 4**	
CORNAS	0.36
GFOLD	0.31
NOISeq	0.19

Average runtimes for the comparisons were about three minutes for NOISeq_nln and NOISeq_tmmnl, one minute for GFOLD, and three seconds for CORNAS_10xless and CORNAS_100xless.

#### Test 3: human sex-specific gene expression

The evaluation of the applicability of CORNAS on real data is based on the human lymphoblastoid cell RNA-Seq data set from Pickrell’s study [24]. In this data set, male and female gender constitute the two phenotype classes, so the true DEG can be determined purely using biological reasoning. The differentially expressed genes were identified as 19 genes with Y chromosome-related expression [5]. Genes that are not differentially expressed on biological grounds include 61 X-inactivated (XiE) genes [25, 26] and 11 housekeeping genes [27].

We randomly chose 100 single female-single male pairs from a total of 725 possible pairs (29 females, 25 males) (Supporting Data), and compared the performance of GFOLD, NOISeq and CORNAS. Our results indicated that NOISeq performed poorly compared to CORNAS and GFOLD, while GFOLD performed slightly better than CORNAS (Fig. 3b, Table 1). However, similar to the compcodeR simulation result, CORNAS called larger DEG sets.

Average runtimes were about two minutes for NOISeq, thirty seconds for GFOLD and ten seconds for CORNAS.

#### Test 4: coverage effects in tissue-specific gene expression data

The Marioni data set [28] consists of RNA-Seq data from human liver and kidney sequenced at two different loading concentrations, 3 pM (high) and 1.5 pM (low). We investigated whether CORNAS would be misled into making DEG calls simply on the basis of differing concentration, when both samples are taken from the same tissue. False positive rates were low in CORNAS, with no DEG calls made for comparisons within the same tissue samples with equal concentrations (Additional file 1: Table S1). However, for samples with different concentrations, GFOLD showed fewer false positives than CORNAS. In all instances, NOISeq returned the highest FPR.

A set of 4863 genes was identified to be uniquely expressed in either human liver or kidney tissues cataloged in the tissue expression database, TISSUES [29]. Again, NOISeq performed poorly compared to CORNAS and GFOLD, while CORNAS performed the best (Fig. 3c, Table 1). For all 12 comparisons between different tissue types, the largest DEG sets were called by CORNAS, and the smallest ones by NOISeq.

Generally for different tissue types, the DEG sets called by NOISeq and GFOLD showed poor overlap, compared to overlaps between GFOLD and CORNAS, and between NOISeq and CORNAS (Additional file 1: Figure S3). CORNAS indicated more unique DEG calls for different tissue types. At the same time, a large percentage of DEG calls from GFOLD or NOISeq were also called by CORNAS.

Average runtimes were about five minutes for NOISeq, thirty seconds for GFOLD, and five seconds for CORNAS.

#### Effect of PCR amplification efficiency on sensitivity

**Fig. 4:**
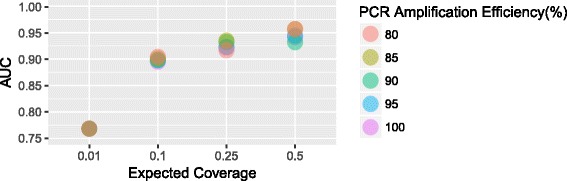
The area under the curve (AUC) of Receiver Operating Characteristic (ROC) analysis for CORNAS runs on data simulated to have 100% 95%, 90%, 85% and 80% PCR amplification efficiencies. The Expected Coverages are the original coverage estimate at 100% PCR amplification efficiency (0.5, 0.25, 0.1 and 0.01)

While we assumed perfect PCR amplification efficiency in building our model, we still evaluated the possible effects of 95%, 90%, 85% and 80% PCR efficiencies on the sensitivity and FPR of CORNAS (details in “Methods” section). CORNAS appears to be robust to small violation of perfect PCR amplification efficiency, as we did not find substantial changes to sensitivity and FPR even at 80% PCR efficiency. The area under the curve (AUC) of the Receiver Operating Characteristic (ROC) graphs of all four tested expected coverages had less than 5% difference (Fig. 4 and Additional file 1: Figure S4).

## Discussion

### CORNAS as a framework for estimating the true gene count

The GP model is being increasingly studied as an alternative to the negative binomial distribution in RNA-Seq count data modelling [30–33]. Here, we demonstrated a chance mechanism that naturally gives rise to the GP as a model for observed gene count data. By relating the parameters of GP to the true gene count and sequencing coverage using RNA sample concentration, we were thus able to determine the posterior distribution of the true gene count. This distribution forms the basis for making DEG calls in unreplicated RNA-Seq experiments.

Currently, the mapped read depth over a gene model of an organism is used to estimate coverage in RNA-Seq experiments. We know that the total amount of mRNA in a sample is not captured in Illumina sequencers, which have a fixed finite saturation amount that can over- or under-represent sample concentrations. The coverage is generally accepted as an under-representation, a limitation that is usually thought to be rectifiable by deep sequencing, which is used to detect genes that have very low mRNA expression [15, 17, 34]. The range of our coverage parameter (between 0 and 1) should cover most practical cases where deep sequencing is not done. We do not recommend the use of CORNAS if the estimated coverage is more than one.

Our study made several assumptions to simplify the model, and one of it is 100% efficient PCR amplification. The effect of PCR amplification efficiency we simulated does indicate that the sensitivity and FPR increases when we over-estimate the coverages, but the change is not detrimentally significant.

The assumption of ideal random cDNA fragment sampling in the current work was made in order to keep the observed count model (hence the posterior distribution) sufficiently simple for us to study the effect of introducing the coverage parameter into the DEG call procedure. Since real RNA-Seq experiments contain library preparation biases, the effect of such biases may be better explored by full sequencing process simulators such as rlsim [35].

A potential source of variation in the observed gene count that was not explicitly handled in our simulation concerns the way different algorithms map the short reads to a reference genome (e.g. using BWA [36], OSA [37], TopHat [38] and Bowtie [39]), and how such mapped reads are quantified (e.g. using HTSeq [40], and Cufflinks [38]). We suggest that variation in the observed gene count due to this source of variation is relatively unimportant, and hence does not severely affect the posterior distribution of the true gene count. Firstly, algorithms that improve the quality of read alignment [41], and thus minimize counting errors, are available. Furthermore, combinations of read-mapper and gene count quantification have been empirically studied, and optimal recommendations are available to obtain the most reliable observed gene count (e.g. OSA + HTSeq as suggested by [42]).

### Robustness of CORNAS

CORNAS showed comparable performance as GFOLD and NOISeq in the compcodeR simulation, despite being based on a different data model for the observed gene counts (i.e. Generalized Poisson vs. Negative Binomial). This finding provides confidence in integrating the CORNAS framework into current RNA-Seq data analysis protocols. Furthermore, despite the fact that the coverages were estimated, and thus subject to errors, both CORNAS settings (10xless and 100xless) showed similar performance on average. CORNAS struck a good compromise between sensitivity, PPV and DEG set size compared to GFOLD and NOISeq. In real world experiments, CORNAS can outperform competing methods when coverage is more reliably ascertained, such as from the Marioni dataset in Test 4.

Without incorporating information from the coverage parameter, traditional methods such as GFOLD and NOISeq for analysing unreplicated RNA-Seq count data are either too conservative, making very few calls but most of which are true positives (GFOLD), or making relatively more false positive calls (NOISeq) under very low coverage scenario (e.g. *b*=0.01) (Fig. 2). On the other hand, we showed that CORNAS controlled the FPR well and had high TPR when coverages are not too small (e.g. *b*≥0.1). Furthermore, if detection of weak fold change difference is of interest, then the fold-change parameter (*ϕ*) can be reduced from 1.5 to, say, 1.0 (details in the “Methods” section). The TPR profiles of CORNAS at fold-change parameter of 1.0 becomes similar to that of NOISeq for weak and strong signals, except when coverage is very low. With increasing true gene count, CORNAS continued to show a general increase in TPR, whereas NOISeq showed decline.

At present, most RNA-Seq experiments do not report an estimate of the actual amount of RNA in the starting material prior to sequencing. As a result, we could only study the effect of correcting the observed gene count using the posterior mean by simulations. Given the encouraging results, researchers may wish to collect information about the coverage parameter in the future to take advantage of CORNAS in the analysis of real RNA-Seq data sets.

A major problem in analysing unreplicated RNA-Seq count data is the lack of effective normalization methods in the absence of biological replicates. Here, we have shown that the Bayesian framework on which CORNAS is based on, avoids the normalization problem by working with the posterior distribution of the gene’s true count. As a result, transcript length information is not required. This makes CORNAS suitable for organisms with incomplete or evolving transcriptome reference data, as new transcript information will not change how true counts are estimated over time. Our results suggest that CORNAS can be used as a means to overcome analytical bottlenecks in experiments with limited replicates and low sequencing coverage leading to DEGs with better prospects of downstream validation using platforms such as quantitative PCR and NanoString nCounter [43]. The result of extending CORNAS to the case of multiple replicates will be published elsewhere.

## Conclusion

We have developed CORNAS (COverage-dependent RNA-Seq), a fast Bayesian method that incorporates a novel coverage parameter to estimate the posterior distribution of the true gene count. Under the CORNAS framework, orthogonal information from sequence coverage that is determined from the concentration of an RNA sample can be used to improve the accuracy of calling DEG. Through simulations and analyses of real data sets, we showed that the performance of CORNAS was comparable or superior to GFOLD and NOIseq in the case of unreplicated RNA-Seq experiments.

## Methods

CORNAS is implemented as an R program and is available for download (https://github.com/joel-lzb/CORNAS). Perl and R scripts for simulation and data analysis work are available at https://github.com/joel-lzb/CORNAS_Supporting_Data. We performed the *in silico* experiments in IBM System x3650 M3 (2x6 core Xeon 5600) machines with 96 GB RAM running on RedHat 6 operating system. Graphs were drawn using ggplot in R [44].

### Simulation of the fragment sampling process and the relationship between coverage and mean/variance of observed counts

Consider a population of *N* cDNA fragments of the same length. In this study, we set *N*=300×10^6^ (300M). We used the following numbers of sequenced reads (*S*): 150 M, 120 M, 75 M, 30 M, 3 M and 0.3 M for simulating coverages (*S*/*N*) of 0.5, 0.4, 0.25, 0.1, 0.01 and 0.001, respectively.

To simulate the process of sampling from the cDNA fragment population, we first indexed each of the *N* cDNA molecule from 1 to *N*. Next, we used the Fisher-Yates shuffle algorithm to shuffle the indices, creating a permutation ***π***=(*π*_1_,*π*_2_,…,*π*_*N*_). The first *S* elements of ***π*** represent the indices of sequenced fragments. For each true gene count *k* from 1 to 100,000, we determined the corresponding observed gene count as

$$X = \sum\limits_{i=1}^{S} I_{(\pi_{i} \leq k)}, $$

where *I*_*A*_ is the indicator function that takes value 1 when the event *A* is true, and 0 otherwise. A total of 2000 iterations were made, and the mean and the variance of the observed gene counts were estimated from them.

Theoretically, the observed counts generated from this process follow a hypergeometric distribution. Thus we are able to calculate the ratio of the mean and variance (*m*) of the hypergeometric distribution for a given coverage *b* as:

6 $$\begin{array}{*{20}l} m &= \frac{S(k/N)}{S(k/N)((N-k)/N)((N-S)/(N-1))} \\ &= \left(\frac{N}{N-k}\right) \left(\frac{N-1}{N-S}\right) \\ &\approx \frac{N}{N-S} \\ &= \frac{1}{1-b} \, \end{array} $$

given *N* is very large (300M), *k* is very much smaller than *N* (≤ 100K) and *b* = *S*/*N*. For sufficiently small *b*, *m*≈1+*b*.

The sampling process naturally leads to a hypergeometric distribution of the observed counts because *N* is finite. However, *N* is large and unknown in practice, hence the need for an approximating distribution that does not have an upper bound (see “Results” section on the GP model).

### Modelling the posterior mean and the posterior variance as functions of coverage

The posterior distribution of true count *k* for an observed count *x* was determined as in Eq. 2. Then we identified the relationship of both the mean and variance of the posterior distribution with the coverage parameter. We first modelled the mean and variance as linear functions such that:

$$\begin{array}{*{20}l} \mu = x \cdot {Gm} + Im\, {;} \quad & \sigma^{2} = x \cdot {Gs} + Is,  \end{array} $$

where the parameters *Gm* and *Gs* are the gradients, and *Im* and *Is* are the intercepts respectively. Then, we fitted models for each of the parameters from simulations at various coverages (Additional file 1: Figure S2). Equations 3 and 4 are the final approximations to model the mean and variance of the posterior distribution as a function of the observed gene count (*x*) and the sequencing coverage (*b*).

### Evaluation of CORNAS

#### Program settings

Two parameters need to be set in CORNAS. The first one is *α*, which is used for determining the lower (1−*α*)/2×100th percentile (*p*_(1−*α*)/2_) and the upper (1+*α*)/2×100th percentile (*p*_(1+*α*)/2_). The second parameter is the fold-change cut-off *ϕ*. To make a DEG call, we require $p^{+}_{(1-\alpha)/2} / p^{-}_{(1+\alpha)/2} \geq \phi $, where the superscript + and − indicate the the posterior distribution with higher and lower mean, respectively. The default settings are *α*=0.99 and *ϕ*=1.5. These values can be changed to make CORNAS more conservative (e.g. increasing *α* and/or *ϕ*), or more liberal (e.g. lowering *α* and/or *ϕ*).

NOISeq was run with a q=0.9 cut-off. GFOLD was run with a 0.01 significance cut-off for fold changes. The expression of a gene was considered up-regulated if the GFOLD value was 1 or greater and down-regulated if the GFOLD value was -1 or smaller.

#### Performance metrics

True positives (TP) are genes known to be differentially expressed between two samples, and are detected as DEG by the methods evaluated. False DEG calls are false positives (FP), while false negatives (FN) are missed true DEG calls. For a DEG call method, its positive predictive value (PPV) is the proportion of calls that are true DEG (TP/(TP+FP)) and its sensitivity is the proportion of true DEG that are called (TP/(TP+FN)). We considered sensitivity and PPV of each method jointly for Tests 2, 3 and 4. The F-score, which is the harmonic mean of sensitivity and PPV, was calculated for each comparison as 2×(*sensitivity*×*PPV*)/(*sensitivity*+*PPV*). The mean F-score per method was reported.

For Test 1, the false positive rate (FPR) is determined from the no-fold change scenario as the true negatives (TN) are explicitly known (FP/(FP+TN)) while the sensitivity is calculated similarly as that in Tests 2, 3 and 4 from the weak and strong effect scenarios.

#### Test 1: detection of differentially expressed genes in simulated true gene count data

For this simulation, three scenarios of biological effects were considered: no fold change (no effect), 1.5-fold change (weak effect), 2-fold change (strong effect). The maximum true counts considered under these three scenarios were 10,000, 6,666 and 5,000, respectively. We assumed that each true gene count was emitted by a gene, so that the set of all true gene counts under all three scenarios corresponded to a total of 21,666 genes. The observed counts for each gene was generated following the procedure described in the simulation of the fragment sampling process. A total of 100 iterations were made to account for sampling variability in observed gene counts. Where gene length information is required for a particular method, we set it at 1000 bases.

#### Test 2: compcodeR simulation

We generated the simulated data set B_625_625 according to the methodology described in the compcodeR paper [23]. The number of DEG constituted 10% of the total number of genes (12,498).

#### Test 3: human sex-specific gene expression

For the Pickrell study consisting of 29 females and 25 males from Nigeria, we used the number of total sequenced reads from the published paper [24] and the RNA-Seq count data from the ReCount database [45]. The sequencing coverage for each sample was calculated as the number of total reads reported divided by the standard 300M cDNA fragment size. For samples with more than one sequencing run, we took the average of the total reads generated.

#### Test 4: coverage effects in tissue-specific gene expression data

In the Marioni data set, the same human liver and kidney samples were sequenced in seven lanes each, with five lanes loaded at an RNA concentration of 3 pM, and another two with 1.5 pM. The 14 lanes were sequenced in two separate runs. To reduce technical variation, we used only data from run 2, where loadings with different concentrations were run under the same conditions and time. We estimated the number of cDNA fragments representing the sample’s transcriptome as the product of the loading concentration, the loading volume (assumed as standard 120 *μ*L), and the Avogadro constant 6.022×10^23^mol^−1^. The set of true DEGs used was identified based on curated information extracted from TISSUES [29] on the 14th of June 2016. We selected 737 human kidney genes and 4,126 human liver genes that have supporting experimental validation results and are identifiable with Ensembl gene ID.

#### Effect of PCR amplification efficiency on sensitivity

The evaluation was conducted with the same dataset used for Test 1. To simulate the effect of PCR amplification efficiency in the study, we recalculated the sequencing coverages for each CORNAS run by reducing the assumed total number of fragments prior to PCR caused by different PCR amplification efficiencies (70%, 49%, 34%, 23% of total fragments for 95%, 90%, 85%, 80% amplification efficiency respectively). Details on how the PCR amplification efficiencies were determined can be found in the Additional file 1. For example, a sample that had perfect amplification but had a sequencing coverage of 0.25 would have 300M fragments prior to PCR and 75M reads produced. Supposed the reads produced remains unchanged, but the PCR amplification efficiency is now 95%, the sequencing coverage estimated will then be 0.36 (75M / (300M × 0.7)). The new coverage is then used in the 0.25 coverage CORNAS run with 95% PCR amplification efficiency. For each coverage, the FPR were calculated from the number of DEG called in the no effect scenario, and the sensitivity was calculated from the DEG called from the strong effect scenario. We generated the Receiver Operating Characteristic (ROC) curves using the ROCR R package [46]. The cut-offs for making a differential expression call were obtained by fixing *α*=0.99 and then varying *ϕ* from 1.5 to 0.75, and by fixing *ϕ*=0.75 and then varying *α* from 0.99 to 0.01.

## Additional file

Additional file 1 Additional results referred in the main text. (PDF 569 kb)

## Abbreviations

cDNA: Complementary DNA
DEG: Differentially Expressed Genes
DNA: Deoxyribonucleic acid
FN: False negative
FP: False positive
FPR: False positive rate
GP: Generalized poisson
mRNA: Messenger RNA
PCR: Polymerase chain reaction
PPV: Positive predictive value
RNA: Ribonucleic acid
TN: True negative
TP: True positive
TPR: True positive rate
