# Supplementary material for: CORNAS: coverage-dependent RNA-Seq analysis of gene expression data without biological replicates
Source: BMC Bioinformatics. 2017 Dec 28;18(Suppl 16):575. doi: 10.1186/s12859-017-1974-4 (PMC5751784; doi:10.1186/s12859-017-1974-4)
Supplement: Additional file 1 — Additional results referred in the main text. (PDF 569 kb) [file 12859_2017_1974_MOESM1_ESM.pdf]

1 RNA-SEQ COVERAGE

1.1 Illumina's sequencing procedure

Illumina's platform is widely used for differential gene expression analyses due to its ability to sequence deeper than 454 at lower costs. While we will not go into great detail, the following are nuances during the library preparation that will need to be accounted for:

Starting material. For an Illumina run using TrueSeq stranded mRNA sequencing library preparation, 1 μg of total RNA is usually needed.

mRNA isolation. Most RNA-Seq studies are conducted on mRNA. Less than 1% of the total RNA survives mRNA isolation (poly-dT beads), including mRNA. Usually, the loss of mRNA in the wash is due to degraded mRNA, i.e. poor total RNA quality.

Fragmentation. This process produces $\approx$ 500 nt long fragments. The process is followed by a size selection procedure which further increases mRNA loss.

cDNA preparation. The next phase is then cDNA preparation with random hexamer priming which introduces priming biases.

PCR. This step is needed to increase the amount of RNA. It is noted that overloading (too concentrated) the flow cell produces no results, and underloading (too diluted) can cause very skewed results. Most cases require PCR, because underloading occurs. Furthermore, the amount is greatly affected by the starting sample concentration, e.g. 200 ng, which isn't the same for all samples. One sample may need PCR, while another does not, so doing PCR for both, will introduce equal duplication events to cancel out comparison bias. However, to reduce duplication bias, the cycle is kept as low as possible, which is generally 14 cycles.

Loading volume for sequencing. The final product of PCR would yield $\approx$ 40 μL of 200 nM (nanoMolar). The amount then gets diluted 20,000 times to a loading amount of 120 μL for the flow cell. The 40 μL is first diluted 100 times to 2-3 nM, and then further diluted 200 times as aliquots.

1.2 What is the total mRNA found in a sample?

To identify a sample's sequencing coverage, we will need to first identify what is the size of the mRNA population to compute the sample's sequenced proportion from. While it is ideal to obtain the number of total mRNA available prior to library preparation, the biases mentioned above make it difficult, if not impossible, to allow accurate estimates of the total mRNA in the sample. We reasoned that the amount of cDNA produced at the step prior to PCR would provide us the most reliable means for computation because:

1. The fragmentation step causes homogeneity of the cDNA molecule sizes.
2. The volume and concentration after PCR is known.
3. The number of PCR cycles is known.

1.3 What is the original amount of cDNA before PCR?

We can calculate this quantity since the cDNA molecules would have similar molecular weights after size selection ($\approx$ 500 nt). The PCR final volume of 40 μL has 200 nM (200 nmol/L) concentration of 500 bp cDNA molecules, which translates to 4.818×10^{12} of cDNA molecules. Assuming complete replication efficiency, a cDNA molecule would be amplified 2^{14} times for 14 cycles of PCR. Therefore the number of cDNA before PCR, in an ideal case where all cDNA are amplifiied, is $\frac{4.818 \times 10^{12}}{2^{14}} = 294,067,382$.

1.4 The true coverage of RNA-Seq experiments

PCR is required to improve the chance of one cDNA to be picked for sequencing by having it copied ten thousand times. The chance of picking the original amount for each cDNA species prior to PCR should be very high if the dilutions are perfectly homogenous after PCR. If we work with the assumptions above as the ideal case, we are able to calculate the coverage of sequencing, based on the number of sequenced reads obtained over the number of cDNAs available before PCR.

2 PCR AMPLIFICATION EFFICIENCY

Define the random variable X which has a beta distribution with mean $\alpha/(\alpha+\beta)$. We can use X to model the deviation from perfect amplification by considering the random variable $2-X$. Let k be the number of PCR cycles, and N_0 the initial number of DNA fragments. Assuming perfect amplification, the number of fragments after k cycles of amplification is

$$S_p = N_0 2^k.$$

If we assume amplification efficacy in each cycle is independent of one another, then the actual number of fragments after k cycles is

$$S_a = N_0 \prod_{i=1}^{k} (2 - X_i).$$

Thus, the expected relative effect of variation in amplification efficiency is given by

$$\begin{aligned}
\mathbb{E}\left(\frac{S_a}{S_p}\right) &= \frac{1}{2^k}\prod_{i=1}^{k}\mathbb{E}(2-X_i) \\
&= \frac{1}{2^k}[\mathbb{E}(2-X_1)]^k \\
&= \left(1-\frac{\alpha}{2(\alpha+\beta)}\right)^k
\end{aligned}$$

For the variance of S_a/S_p, we have

$$\begin{aligned}
\mathrm{Var}\left(\frac{1}{2^k}\prod_{i=1}^{k}(2-X_i)\right) &= \mathrm{Var}\left[\prod_{i=1}^{k}\left(1-\frac{X_i}{2}\right)\right] \\
&= \mathbb{E}\left[\prod_{i=1}^{k}\left(1-\frac{X_i}{2}\right)\right]^2 - \left[\prod_{i=1}^{k}\mathbb{E}\left(1-\frac{X_i}{2}\right)\right]^2 \\
&= \left\{\mathrm{Var}\left(1-\frac{X_1}{2}\right) + \left[\mathbb{E}\left(1-\frac{X_1}{2}\right)\right]^2\right\}^k - \left(1-\frac{\alpha}{2(\alpha+\beta)}\right)^{2k} \\
&= \left[\frac{\alpha\beta}{4(\alpha+\beta)^2(\alpha+\beta+1)} + \frac{\beta^2}{4(\alpha+\beta)^2}\right]^k - \left(1-\frac{\alpha}{2(\alpha+\beta)}\right)^{2k} \\
&= \frac{[\beta(\alpha/(\alpha+\beta+1)+\beta)]^k - (\alpha+2\beta)^{2k}}{[4(\alpha+\beta)^2]^k}
\end{aligned}$$

The following table gives the expected proportion of fragments under a beta model of amplification variation relative to perfect amplification.

α	β	$\mathbb{E}(S_a/S_p)$	$\mathbb{E}(S_a/S_p) \pm$ 2SD
5	95	0.70	0.64 - 0.76
10	90	0.49	0.43 - 0.55
15	85	0.34	0.29 - 0.38
20	80	0.23	0.19 - 0.27

3 SUPPLEMENTARY FIGURES AND TABLE

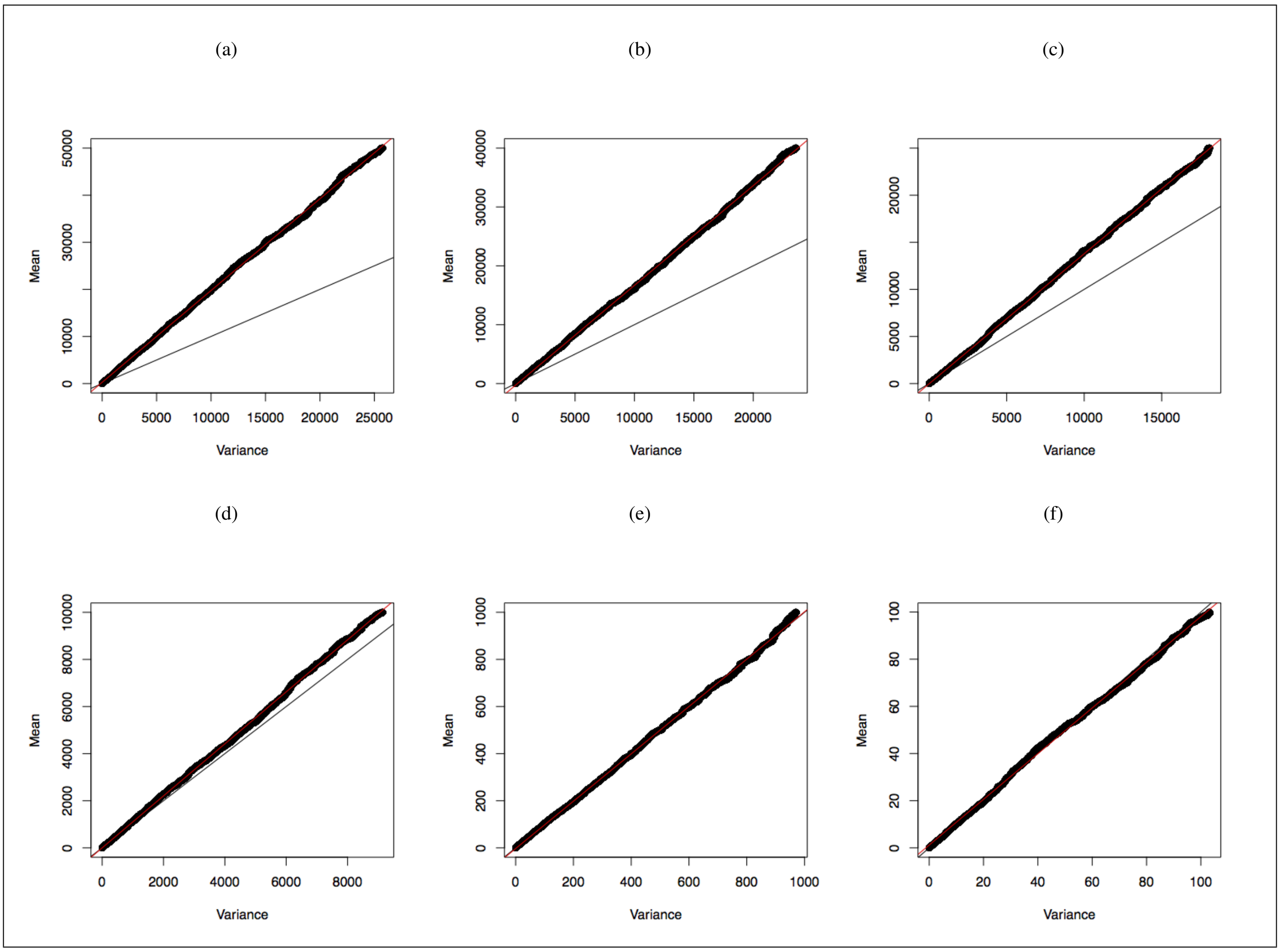

Figure S1: Mean vs variance of observed counts in 2,000 replicates for the following coverages (a): 0.5X, (b): 0.4X, (c): 0.25X, (d): 0.1X, (e): 0.01X, (f): 0.001X. The black line is where mean is equal to variance. The red line is the fitted linear model.

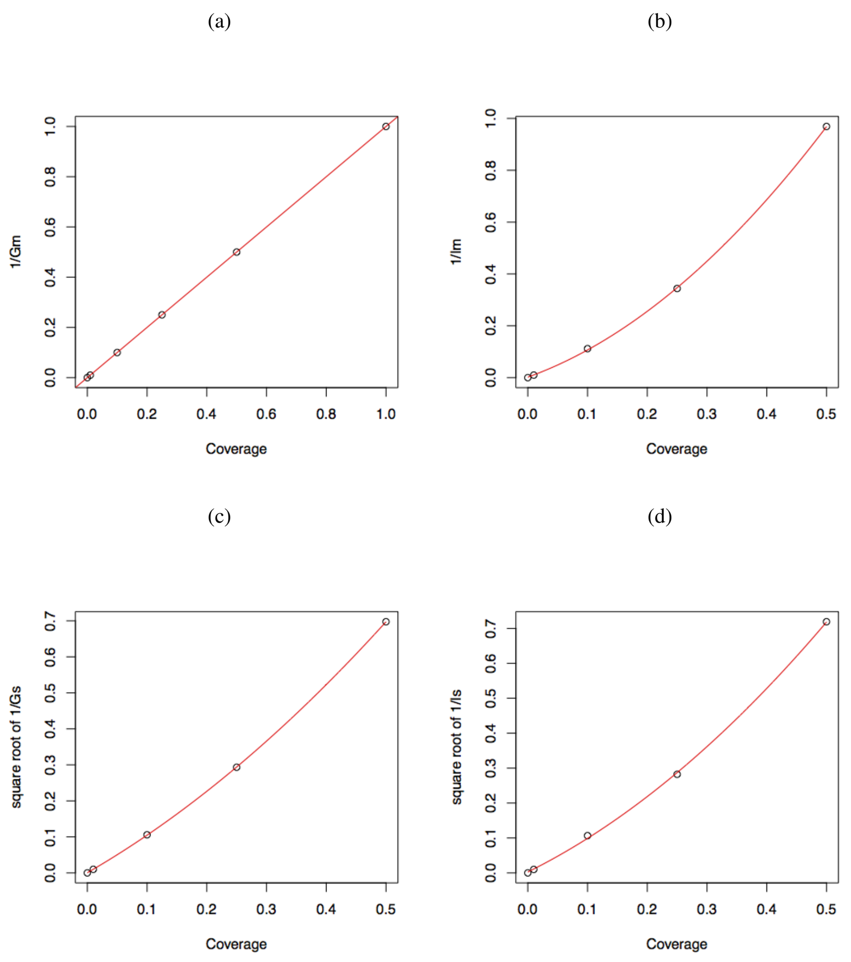

Figure S2: The relationship of the sequencing coverage with the slope and intercept parameters of linear models of the posterior mean and posterior variance; where (a): Gm, (b): Im, (c): Gs, (d): Is are respectively modelled in the equations of Section 5.3. The open circles represents the simulated data used to estimate the model.

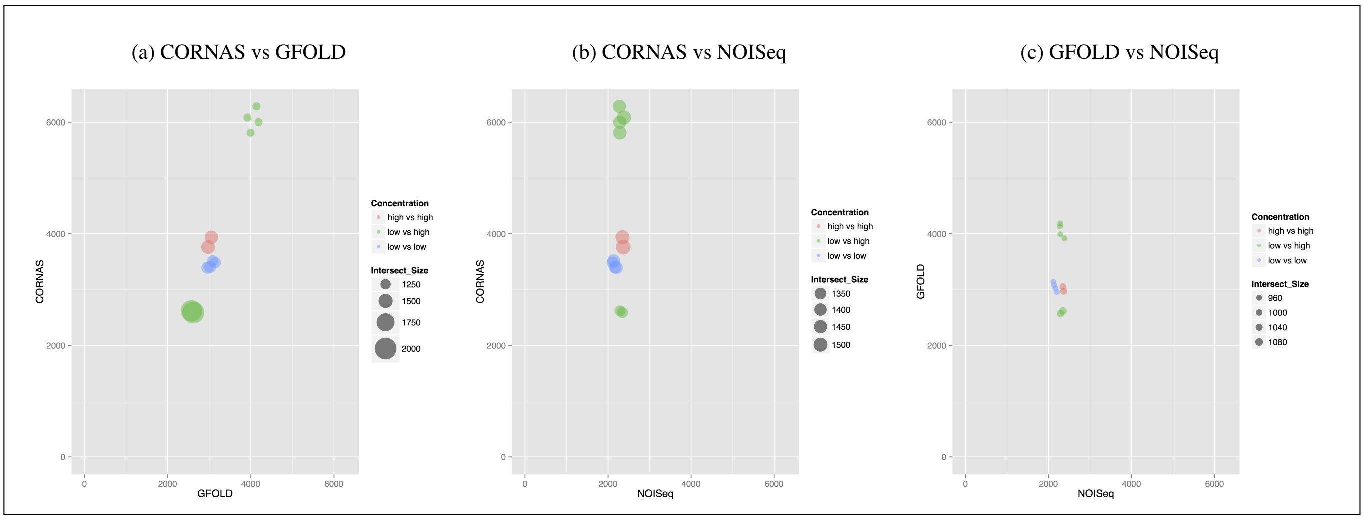

Figure S3: DEG set agreement between methods in analysing 12 comparisons between three human liver and four kidney samples. The axes represents the number of DEG called for each method, while the circle size approximates the intersect size. Two types of sample loading concentrations were used, 3 pM (high) and 1.5 pM (low). Details can be found in Table S1.

Concentration	Type	Sample A	Sample B	NOISeq	GFOLD	CORNAS
low vs low	same tissue	R2L4Kidney	R2L8Kidney	275	0	0
high vs high	same tissue	R2L2Kidney	R2L6Kidney	333	1	0
low vs high	same tissue	R2L4Kidney	R2L2Kidney	329	0	42
low vs high	same tissue	R2L8Kidney	R2L2Kidney	335	0	29
low vs high	same tissue	R2L4Kidney	R2L6Kidney	356	1	124
low vs high	same tissue	R2L8Kidney	R2L6Kidney	325	0	82
low vs high	same tissue	R2L1Liver	R2L3Liver	324	0	105
low vs high	same tissue	R2L7Liver	R2L3Liver	308	1	46
low vs low	same tissue	R2L1Liver	R2L7Liver	307	1	0
low vs high	different tissue	R2L4Kidney	R2L3Liver	2347	2616	2588
low vs high	different tissue	R2L8Kidney	R2L3Liver	2288	2570	2619
high vs high	different tissue	R2L3Liver	R2L2Kidney	2366	2972	3761
high vs high	different tissue	R2L3Liver	R2L6Kidney	2348	3051	3937
low vs low	different tissue	R2L1Liver	R2L4Kidney	2113	3143	3484
low vs low	different tissue	R2L1Liver	R2L8Kidney	2135	3083	3517
low vs high	different tissue	R2L1Liver	R2L2Kidney	2285	4185	6000
low vs high	different tissue	R2L1Liver	R2L6Kidney	2273	4134	6284
low vs low	different tissue	R2L7Liver	R2L4Kidney	2202	2956	3392
low vs low	different tissue	R2L7Liver	R2L8Kidney	2163	3022	3405
low vs high	different tissue	R2L7Liver	R2L2Kidney	2283	3993	5810
low vs high	different tissue	R2L7Liver	R2L6Kidney	2385	3918	6083

Table S1. DEG calls made by NOISeq, GFOLD and CORNAS between two samples from Marioni's data. The sample combinations consisted of two human tissue types (Liver and Kidney) with two loading concentrations, 3 pM (high) and 1.5 pM (low).

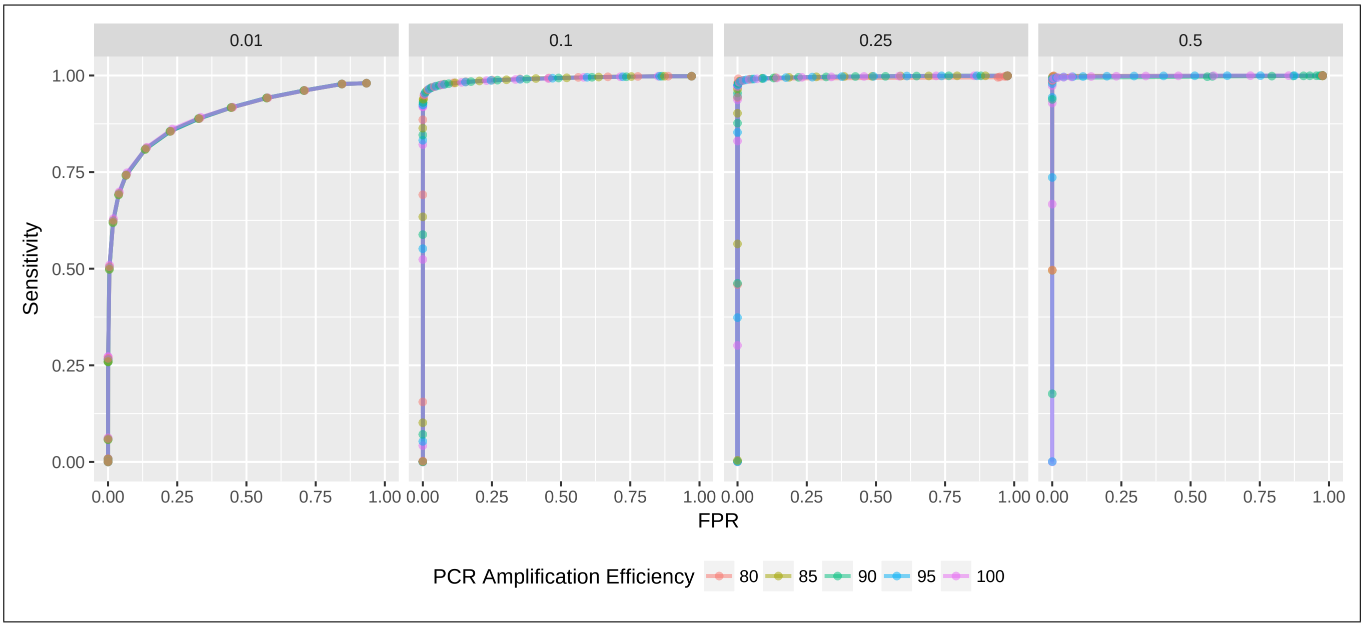

Figure S4: CORNAS sensitivity against false positive rates (FPR) for data simulated to have 100% 95%, 90%, 85% and 80% PCR amplification efficiencies, facetted according to the expected coverage estimates at 100% PCR amplification efficiency (0.5, 0.25, 0.1 and 0.01).
